# Post-operative complications of gastric cancer surgery: female gender at high risk

**DOI:** 10.1111/j.1365-2354.2008.01036.x

**Published:** 2009-03

**Authors:** BK SAH, ZG ZHU, XY WANG, QM YANG, MM CHEN, M XIANG, J CHEN, M YAN

**Affiliations:** Department of General Surgery, Rui Jin Hospital, Shanghai Jiao Tong University, School of Medicine. Shanghai Institute of Digestive SurgeryShanghai, China

**Keywords:** post-operative complications, gastric cancer, POSSUM, gender difference

## Abstract

We applied physiological and operative severity score for the enumeration of morbidity and mortality (POSSUM) to evaluate overall surgical outcome and investigated the role of gender for early post-operative complications in gastric cancer surgery. The data from a total of 357 patients of gastric cancer were analysed by univariate and multivariate analysis. Post-operative complications were recorded according to definition of POSSUM. Post-operative complications of male and female patients were compared separately. The observed to estimated morbidity ratio (O:E) was 1.01. Among the pre-operative variables, patient gender was one of the independent risk factors for a higher rate of post-operative complications (risk ratio 1.777, *P* = 0.024). Post-operative complication was significantly higher in female patients. Similarly, post-operative length of stay was significantly longer and more severe complications were observed in female patients (*P* = 0.03). In conclusion, POSSUM system is a valid algorithm for risk-adjusted surgical audit. We conclude that a patient's gender influences the early post-operative complications after gastric cancer surgery. A detailed understanding on disparity of early post-operative complications between men and women may provide valuable information to improve surgical outcome of gastric cancer. However, results of this study need further confirmation by a prospective study involving a larger cohort.

## INTRODUCTION

### Complications of gastric cancer surgery

Post-operative complications of gastric cancer surgery are still considered higher, which is mainly attributed to the extent of surgery and technical inexperience (McCulloch *et al.* 2005; Pedrazzani *et al.* 2007). The extent of surgery for gastric cancer is highly heterogeneous; however, there has been consensus that surgery only cannot improve the survival rate (Hartgrink *et al.* 2004; Yasuhiro 2007). The rate of early post-operative complications is as important as the rate of survival. Therefore, post-operative complications must be considered seriously while practicing surgery for gastric cancer.

### Standard system for surgical audit

Simple collection of complication numbers alone is not sufficient to reflect treatment quality as, to compare morbidity and mortality data directly, the original populations must be identical (Whiteley *et al.* 1996). The physiological and operative severity score for the enumeration of morbidity and mortality (POSSUM) is a valid system for risk-adjusted auditing which has been extensively used in various surgical specialties (Copeland *et al.* 1991; Whiteley *et al.* 1996; Jones & de Cossart 1999; Neary *et al.* 2003). Complication of different groups of patients can be compared directly on the basis of physiological score (PS) and operative severity score (OSS) components of POSSUM system. Physiological and operative severity score for the enumeration of morbidity and mortality has definitions for post-operative complications, which can be used as standard definition for auditing.

### Gender-based difference in surgical outcome

There were mixed reports of gender-based differences in surgical outcome; sex is hypothesized to be a determinant of the immunologic variability after severe traumatic and surgical stress and, at least in animal models, accounts for differences in outcomes (Homo-Delarche *et al.* 1991; Wichmann *et al.* 1996; Angele *et al.* 1997; Zellweger *et al.* 1997; Knoferl *et al.* 2002; Yokoyama *et al.* 2003). Male gender has been associated with anastomotic leakage, in colorectal surgery (Branagan & Finis 2005), as well as in rectal surgery (Rullier *et al.* 1998; Law *et al.* 2000; Yeh *et al.* 2005). However, in our review, we found no such reports on gastric cancer surgery. We analysed the risk factors for early post-operative complications after gastric cancer surgery and applied POSSUM for risk-adjusted auditing to evaluate overall surgical outcome.

## METHODS

The data of 357 gastric cancer patients were collected directly by comprehensive review of the original records (Table 1). Eight patients were excluded owing to incomplete data. However, there were no deaths or major complications in the excluded cases according to information provided by the hospital database. The ratio of the incidence of gastric cancer in male and female patients was approximately 7:3. All the patients with early and resectable advanced gastric cancer (without significant distant metastases) underwent gastrectomies with D2 lymphadenectomy; few advanced stage patients were performed palliative surgery. Because of inadequate numbers of harvested lymph nodes, we could not document all the pathological data according to TNM classification.

**Table 1:** Demographic data of the patients

Details		Male	Female
Physiological score	Number of cases (%)	252 (70.6)	105 (29.4)
	Median age, year	59	58
	Median PS	15	16
Operative severity score	Median OSS	18	18
Diagnosis	Primary gastric cancer	244	102
	Gastric stump cancer†	8	3
Number of procedures (%)	Partial gastrectomy	185 (73.4)	79 (75.2)
	Total gastrectomy	54 (21.4)	20 (19)
	Gastrojejunostomy*	13 (5.2)	6 (5.7)

* Palliative surgery, tumour not resected.

† Including recurrent gastric cancer.

POSSUM data were collected and calculated as described by the original authors (Copeland *et al.* 1991). Homogeneity of both genders was compared by PS and OSS components of POSSUM system.

### Definition of morbidity

The only endpoint of this study was analysis of in-patients' morbidity or mortality. According to general rule of the hospital, all the patients were only discharged after smooth recovery and removal of suture.

Morbidity was recorded according to definition of POSSUM (Copeland *et al.* 1991). Any observed complication, which was not defined in POSSUM, was categorized as ‘innominate’ in this study. Such complications were only recorded for the overall auditing of all post-operative complications and did not affect the POSSUM calculation.

All complications were further stratified according to Rui Jin Hospital system for classification of complications (Table 2). Patients having multiple complications were grouped into the highest level of their respective complications, e.g. a patient with minor, moderate and severe complication was categorized into the severe group.

**Table 2:** Rui Jin Hospital classification of complications

Minor	Infection: superficial wound infection, deep infection*, chest infection*, urinary infection, septicaemia, pyrexia of unknown origin*Miscellaneous: superficial wound dehiscence, wound haemorrhage, impaired renal function*, deep venous thrombosis*, hypotension
Moderate	Infection: deep infection†, chest infection†, pyrexia of unknown origin†Miscellaneous: deep wound dehiscence, impaired renal function†, deep venous thrombosis†Innominate†
Severe	Systemic: cardiac failure, respiratory failure, pulmonary embolus, hypotension‡, death Surgical: deep haemorrhage, deep infection‡, anastomotic leak Innominate: complications with post-operative stay >30 days

* Post-operative ≤ 15 days.

† Post-operative > 15 days.

‡ Requiring laparotomy.

## STATISTICAL ANALYSIS

spss 13.0 (SPSS Inc., Chicago, IL) statistics tool was used for statistic calculation. Univariate analysis was performed using the chi-square test. Multivariate analysis was performed using the logistic regression model for analysis of risk factors for post-operative complications. Post-operative complication rates were statistically analysed by chi-squared test. A *P*-value of less than 0.05 was considered statistically significant.

We applied exponential analysis method for POSSUM system (Wijesinghe *et al.* 1998). We calculated O:E ratio of morbidity to give risk-adjusted morbidity. An O:E ratio less than one implies a performance that was better than expected and an O:E ratio greater than one indicates a performance that was worse than expected. Test of normality for age, PS and OSS were checked by Shapiro–Wilk method and non-parametric statistic method (Mann–Whitney U) were applied to test the homogeneity of these variables.

## Results

Univariate analysis revealed that the pre-operative variables, patient gender, different surgical units, extent of malignancy, mode of operations and intra-operative blood loss were significantly associated with a higher rate of post-operative complications. There were no significant associations with age, anaemia, hypoalbuminaemia, hyperglycaemia or other variables in POSSUM (Table 3). Among the factors selected from the univariate analysis, patient gender (risk ratio 1.777, *P* = 0.024), surgical units B (risk ratio 2.963, *P* = 0.007) and E (risk ratio 2.947, *P* = 0.008), and intra-operative blood loss (risk ratio 2.598, *P* = 0.000) were independent risk factors for post-operative complications, while extent of malignancy and mode of operations were not (Table 4).

**Table 4:** Multivariate analysis of risk factors for early post-operative complications

Variables	Odd ratio	95% CI	*P*-value
Gender			
Male	1		
Female	1.777	1.080–2.924	0.024
Wards			
A	1		
B	2.963	1.348–6.516	0.007
C	1.004	0.524–1.926	0.990
D	0.842	0.298–2.384	0.746
E	2.947	1.321–6.574	0.008
Blood loss			
<500 mL	1		
>500 mL	2.598	1.602–4.184	0.000

CI, confidence interval.

**Table 3:** Univariate analysis of risk factors for early post-operative complications

	Complication		%	
Variables	Yes	No	complication rate	*P*-value
Gender				0.038
Male	88	164	34.9	
Female	49	56	46.7	
Age (years)				0.125
<60	71	125	36.2	
61–70	33	61	35.1	
>70	33	34	49.3	
Anaemia				0.190
Present	20	22	47.6	
Absent	117	198	37.1	
Hypoalbuminaemia				0.991
Present	15	24	38.5	
Absent	122	196	38.4	
Hyperglycaemia				0.532
Present	11	22	33.3	
Absent	126	198	38.9	
Intraoperative blood loss				0.000
<500 mL	71	160	30.7	
>500 mL	66	60	52.4	
Malignancy				0.034
Primary tumour	42	92	31.3	
Metastases	95	128	42.6	
Operation type				0.003
Partial gastrectomy	89	175	33.7	
Total gastrectomy	41	33	55.4	
Gastrojejunostomy	7	12	36.8	
Units				0.001
A	22	39	36.1	
B	31	24	56.4	
C	49	108	31.2	
D	7	23	23.3	
E	28	26	51.9	

### Risk-adjusted morbidity

Overall 137 patients were observed to have post-operative complications (including death). Exponential analysis of overall patient showed O:E ratio of 1.01 (137:135). A chi-square test showed a significant lack of fit (χ^2^ = 2.15, d.f. = 4, *P* = 0.71), i.e., observed values were not significantly different from predicted value.

**Figure 1:**
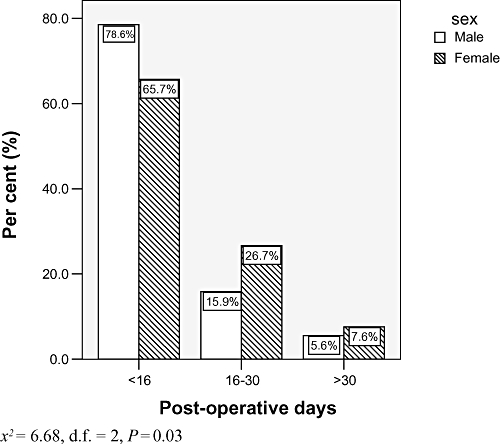
Distribution of patients according to post-operative duration.

**Figure 2:**
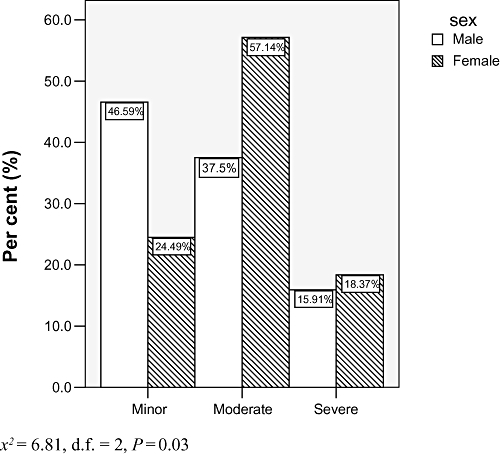
Distribution of patients according to complication type.

Patients of both genders were uniform as there was no significant difference in their PS (*P* = 0.07) and OSS (*P* = 0.08), but there was a significant difference in complication rate between male and female patients (*P* = 0.03). Overall post-operative complication was higher in female patients, especially mortality and infection rate which have odds ratio of 3.67 and 1.36 respectively. Similarly, the duration of post-operative stay was significantly longer (Fig. 1) in female patients (*P* = 0.03) and they had more severe complications (Fig. 2) than male patients (*P* = 0.03). Details concerning the complications in both genders are shown in Tables 5 and 6. The number of complications is not equal to number of patients because multiple complications were possible in a single patient. More interestingly, after stratification according to age (Table 7), we observed significant difference in complication rate between male and female patients of a particular age period (46 and 55 years).

**Table 6:** Details of innominate complication

Complications	Frequency
Pleural effusion	13
Seroperitoneum	11
Suspicious anastomotic leak	11
Gastroplegia / Enteroplegia	7
Continuous pyrexia	5
Pancreatic fistula	2
Biliary fistula	1
Recurrent asthma	1
Urinary retention	1
Refractoriness hiccup	1

**Table 7:** Complication rate

		Complication rate(%)		
Age (years)	Number of patients	Female	Male	*P*-value
≤45	40	40	35	0.74
46–55	87	52	24.2	0.01
56–65	114	41.4	37.6	0.72
>65	116	51.6	40	0.26
Total	357	46.7	34.9	0.03

**Table 5:** Details of complications

		Count		
Complication		Female	Male	Odds ratio (95% CI)
Overall		49	88	1.63 (1.02, 2.59)
Haemorrhage	Wound	0	1	
	Deep	0	2	
Wound dehiscence	Superficial	0	3	
	Deep	0	0	
Anastomotic leak Infection		4	9	1.07 (0.32, 3.55)
	Wound	1	2	1.36 (0.83, 2.21)
	Deep	5	6	
	PUO	18	32	
	Septicaemia	1	0	
	Chest	15	31	
	Urinary	7	8	
System failure	Renal	2	4	1.20 (0.29, 4.91)
	Respiratory	3	3	
	Cardiac	3	3	
	Hypotension	4	0	
Innominate		17	27	1.61 (0.83, 3.09)
Death		3	2	3.67 (0.60, 22.32)

CI, confidence interval; PUO, pyrexia of unknown origin.

## DISCUSSION

### Treatment strategy of gastric cancer

The extent of surgery for gastric cancer is controversial (Swan & Miner 2006), and history witnessed tremendous changes in treatment strategy of this malignant disease. Despite differences in surgical treatment pattern between Japan and western countries, especially on the concept of radical dissection with lymphadenectomy (Hartgrink *et al.* 2004; Sano *et al.* 2004; Swan & Miner 2006), several modes of surgical interventions have been practiced over the past 40 years to cope with the huge prevalence of gastric cancer disease in the region. The poor understanding of its biological behaviour in the past made gastric cancer surgery highly heterogeneous in practice and resulted in different rates of morbidity and mortality according to the extent of surgery, especially in inexperienced hands (Lo *et al.* 2002; Hartgrink *et al.* 2004; Pedrazzani *et al.* 2007). Gradually, the high morbidity and mortality of gastric cancer surgery undermined the role of surgical treatment and the better understanding of oncological behaviour of the disease, compelled aggressive surgeons to review the result and move downward in the extent of surgery (Dicken *et al.* 2005). And if we analyse the trend of extent of surgery, it has been sloping down to the optimum extent and multimodal treatment strategies have been accepted even in Japan (Takashi *et al.* 2005; Yasuhiro 2007). Therefore, more researches on post-operative complications of gastric cancer surgery may play vital role in optimizing surgical extent.

### Interpretation of POSSUM analysis

Physiological and operative severity score for the enumeration of morbidity and mortality exhibited better performance of our unit, as observed morbidity was close to estimated morbidity by POSSUM. However, most of the complications were minor or moderate. And in general, most of these complications are considered of less clinical importance. As POSSUM was originally devised to fit all general surgery cases, including minor surgery, therefore, it accounts for very minor complications, too; but for major operations, many of these complications are negligible and if we account for these complications, definitely morbidities will be higher. Besides, POSSUM has no definition for some specific complications, which is common after gastrointestinal surgery, like pancreatitis, ileus, enteroplegia, pleural effusion etc, which also should be accounted as they extend the post-operative duration of stay in hospital. However, this is not a problem as long as standardized documentation is practiced for POSSUM system and the result can be taken as reference to improve patient care even though it seems to be higher. And a thorough record of all other complications can be kept separately, as we mentioned ‘innominate’ in this study.

### Risk factors for post-operative complications

Univariate and multivariate analysis identified patient gender, particular surgical units and intra-operative blood loss as risk factors for early post-operative complications. However a larger cohort of patients is necessary to support these conclusions. The poor surgical outcome of some units is attributed to poor surgical experience of a particular surgical unit (Sah *et al.* 2008). The role of intra-operative blood loss as a risk factor for post-operative complications has been previously reported by other authors and may be related to blood transfusion, which is said to be associated with immunologic disorders (Takahashi *et al.* 2000; Yasuda *et al.* 2001; Alves *et al.* 2002). Other factors such as age, anaemia, hypoalbuminaemia and hyperglycaemia, which are generally considered to be associated with higher complication rates, did not appear to be risk factors in this study. Further prospective studies are needed to support these data.

### Role of gender disparity on post-operative complications

Although data from animal studies strongly suggest that male gender is a risk factor for an adverse outcome, clinical data are conflicting. Clinically, male gender is an independent risk factor for the development of nosocomial bloodstream infection (Pittet *et al.* 1997), and has been associated with in-hospital mortality in septic surgical patients (Schroder *et al.* 1998). A review (Napolitano *et al.* 2001) of 18 792 patients with blunt trauma found no relationship between gender and mortality. On the other hand, female gender is found to be an independent predictor of mortality in patients with enterococcus bloodstream infections (Stroud *et al.* 1996). Women, in addition, may have a higher mortality among patients with necrotizing soft tissue infection (Elliott *et al.* 1996). Although most studies suggest an increased susceptibility to infectious complications among men, they generally demonstrate a higher mortality rate for women from infections and sepsis (Lephart *et al.* 1987; Wichmann *et al.* 1996; Majetschak *et al.* 2000; Schneider *et al.* 2000), but again, this is not universal (Angele *et al.* 1997; Schroder *et al.* 2000; Martin *et al.* 2003).

There were also some studies, which demonstrated altered level of sex hormones were related with post-operative complications rather than gender. In particular, progesterone in males and testosterone in females had an impact on survival. In both genders, higher 17β-estradiol levels were clearly associated with shorter survival times (Angstwurm *et al.* 2005).

The uncertainty regarding the role of sex in influencing outcomes in humans may simply result from the clinical, phenotypic and genetic diversity among populations and the difficulty in detecting a difference due to these confounders. However, whether males and females respond differently to trauma or surgical insult, either in terms of the host response or the eventual outcome, is critically important for both clinical care and the design of future researches on anti-infective treatments or immuno-biological regulators.

In our study, we observed that the complication rate of female patients of a particular period of age (46–55 years) was more than two times higher than male patients of the same age. And this is the average age period of menopause in women, which is also the period of sex hormone instability. The future study can be focused on the investigation of sex steroids, which may be the possible cause for the gross difference in complication rate between two genders.

## CONCLUSION

Gastric cancer surgery is highly heterogenous in its extent and post-operative complication is still higher. The pre-operative variables of surgical unit, gender and intra-operative blood loss were independent risk factors for early post-operative complications in gastric cancer surgery. However, a prospective study involving a larger cohort is necessary to confirm this result. Physiological and operative severity score for the enumeration of morbidity and mortality system, along with stratification of complications, is a valid algorithm to analyse post-operative complications in gastric cancer patients. In our observation, overall post-operative complication was higher in female patients, especially the mortality and infection rate. A detailed understanding of any such disparity between men and women may provide valuable information for improving surgical outcomes of gastric cancer surgery. If differences do indeed exist, it is possible that future studies might have to stratify for gender and for infection source, and immunologic phenotype. Different mode or management of therapy will become necessary for different sexes.
